# An Early Miocene bumble bee from northern Bohemia (Hymenoptera, Apidae)

**DOI:** 10.3897/zookeys.710.14714

**Published:** 2017-10-19

**Authors:** Jakub Prokop, Manuel Dehon, Denis Michez, Michael S. Engel

**Affiliations:** 1 Department of Zoology, Charles University, Viničná 7, CZ-128 44 Praha 2, Czech Republic; 2 Laboratory of Zoology, Research Institute of Biosciences, University of Mons, Place du Parc 20, 7000 Mons, Hainaut, Belgium; 3 Division of Entomology, Natural History Museum, and Department of Ecology & Evolutionary Biology, 1501 Crestline Drive – Suite 140, University of Kansas, Lawrence, Kansas 66045, USA; 4 Division of Invertebrate Zoology, American Museum of Natural History, Central Park West at 79th Street, New York, New York 10024-5192, USA

**Keywords:** Anthophila, Apoidea, *Bombus*, Burdigalian, geometric morphometrics, Neogene

## Abstract

A new species of fossil bumble bee (Apinae: Bombini) is described and figured from Early Miocene (Burdigalian) deposits of the Most Basin at the Bílina Mine, Czech Republic. *Bombus trophonius* **sp. n.**, is placed within the subgenus Cullumanobombus Vogt and distinguished from the several species groups therein. The species is apparently most similar to the Nearctic B. (Cullumanobombus) rufocinctus Cresson, the earliest-diverging species within the clade and the two may be related only by symplesiomorphies. The age of the fossil is in rough accordance with divergence estimations for *Cullumanobombus*.

## Introduction

**Figure 1.**
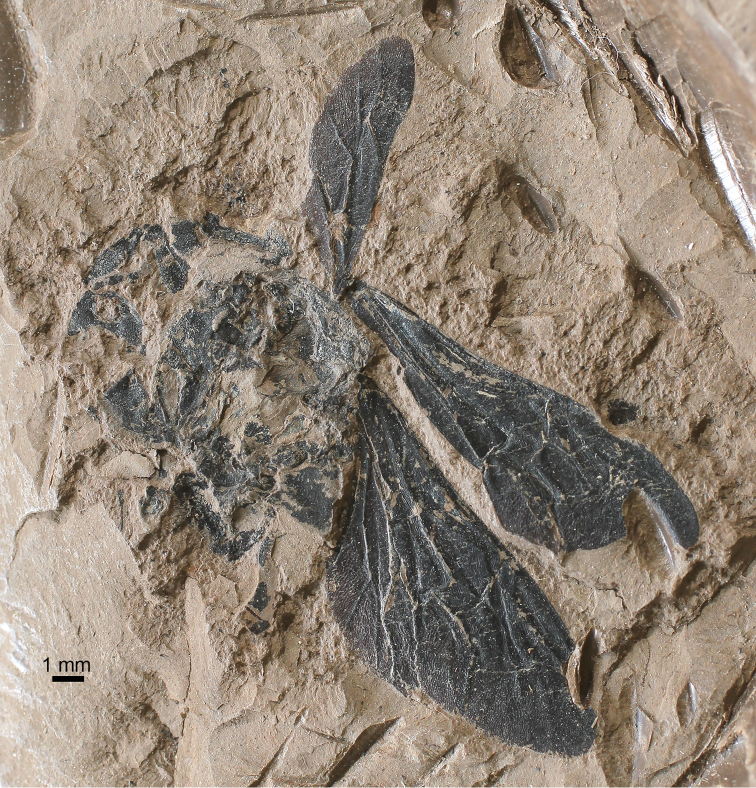
Photograph of holotype of Bombus (Cullumanobombus) trophonius, sp. n., from the Early Miocene of Bílina Mine in northern Bohemia, Czech Republic.

Bumble bees (Bombini: *Bombus* Latreille) are among the most recognized and studied of all bees, second only to the honey bees (Apini: *Apis* Linnaeus) and perhaps tied with the stingless bees (Meliponini). These robust, densely setose, and variably colored species are mainly found in colder temperate regions (Rasmont et al. 2015), and are distributed throughout the Americas, across the Palearctic and Oriental Regions, but are characteristically absent from Africa and Australia (Michener 2007). Together with the orchid bees (Euglossini) and the aforementioned Apini and Meliponini, bumble bees represent one of the four surviving tribal lineages of the corbiculate Apinae (Engel 2001a, Michener 2007). Varied extinct lineages representing stem groups or breaking the otherwise long branches between our modern corbiculates have been discovered from the Paleogene (Cockerell 1908, Engel 1998a, 2001a, Wappler and Engel 2003, Patiny et al. 2007, Engel et al. 2013, 2014), and some of these reveal that the bombine habitus is overall generalized and plesiomorphic for the Corbiculata (*e.g*., Engel 2001a). These extinct clades are also the fossils for which the most information has been accumulated regarding their pollen-collecting behaviors (Wappler et al. 2015, Grímsson et al. 2017). While controversy remains regarding their relationship to either Meliponini or Meliponini + Apini (*e.g*., Michener 1990, Schultz et al. 1999, 2001, Engel 2000a, 2001b, Noll 2002, Cardinal and Packer 2007, Kawakita et al. 2008, Kwang et al. 2017), the 263 extant species of Bombini are likely a comparatively young, monophyletic crown group at the apex of an otherwise older lineage diverging from a common ancestor with meliponines and apines sometime in the latest Cretaceous (Engel 2000, 2001a), leaving a ghost record of stem groups between this divergence and perhaps the Early to mid-Eocene. It is possible that the origin of the crown group for bumble bees could have been associated with a global cooling event that occurred during the mid-Eocene (Hansen et al. 2013, Pound and Salzmann 2017). In fact, this same pattern seems to be true also for euglossines (crown group perhaps of Eocene-Oligocene age) and perhaps apines (latest Eocene or earliest Oligocene age), while crown-group meliponines extend back to the Maastrichtian (Michener and Grimaldi 1988, Engel 2000b). In general, the geological history of the corbiculate bees encompasses one of the more extensive records of fossils among the Apoidea (Michez et al. 2012), with diverse representatives spanning the Cenozoic for the highly eusocial Apini (Engel 1998b, 1999a, 2006, unpubl. data, Engel et al. 2009, Kotthoff et al. 2011, 2013) and Meliponini (Michener 1982, Camargo et al. 2000, Engel 2001a, unpubl. data, Greco et al. 2011, Engel and Michener 2013a, 2013b). Fossils of the communal or solitary Euglossini (Engel 1999b, 2014, Hinojosa-Díaz and Engel 2007) and the primitively eusocial Bombini (Michez et al. 2012, Wappler et al. 2012) are less common, and for this reason are of greater interest when new material becomes available. It is in this context that we provide here a descriptive account for a fossil *Bombus* from the Early Miocene of northern Bohemia (Fig. 1), representing an early record of the subgenus Cullumanobombus Vogt. We provide this description here so that the species’ name might be available for use in a forthcoming work on the general review of fossil record of Bombini (Dehon et al. in prep.).

## Material and methods

**Figures 2–3.**
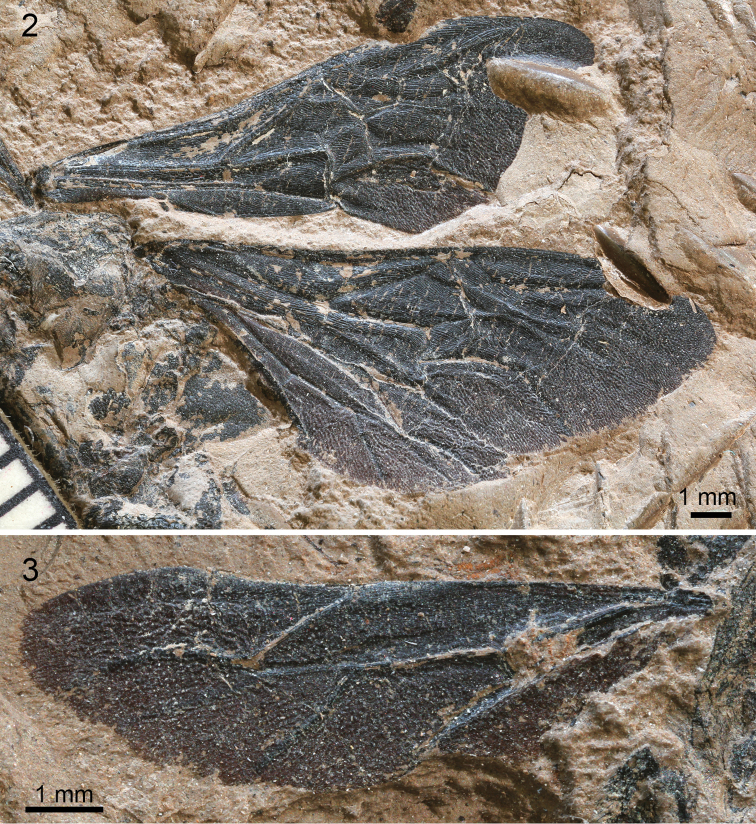
Photographs of wings of holotype of Bombus (Cullumanobombus) trophonius, sp. n. **2** Left forewing and right forewing and hind wing **3** Right hind wing.

**Geological setting.** The Early Miocene coal seam overlaying deposits of the Most Basin at Bílina Mine represents one of the classic paleontological localities in northern Bohemia, studied intensively since the 19^th^ century. The depositional environment and stratigraphy of the upper coal seam deposits at Bílina Mine have been summarized by Kvaček et al. (2004) and updated by Pešek et al. (2014), while the age of the primary insect-bearing layers within the Holešice Member corresponds to the early Burdigalian, from 18–20 Ma (Shrbený et al. 1994, Rajchl et al. 2009). The locality at the time was characterized by a subtropical/warm temperate and temperate climate (Kvaček et al. 2004). The insect fauna at Bílina Mine includes more than 350 specimens of terrestrial and aquatic groups assigned to 31 families in 11 orders (*e.g*., Prokop and Nel 2000, Prokop 2003, Fikáček et al. 2008), with specimens of Hymenoptera, particularly ants, being most prevalent (Prokop and Nel 2003, Wappler et al. 2014). In addition, the overlaying deposits at Bílina Mine have been studied intensively for their remarkably well-preserved record of plant-arthropod interactions (*e.g*., Prokop et al. 2010, Knor et al. 2012, 2013). The bumble bee described here is preserved in a fine clay overlaying the coal seam, and has become carbonized, thus the chitinous integument is modified by the process of fossilization (Figs 1–3).

**Figures 4–6.**
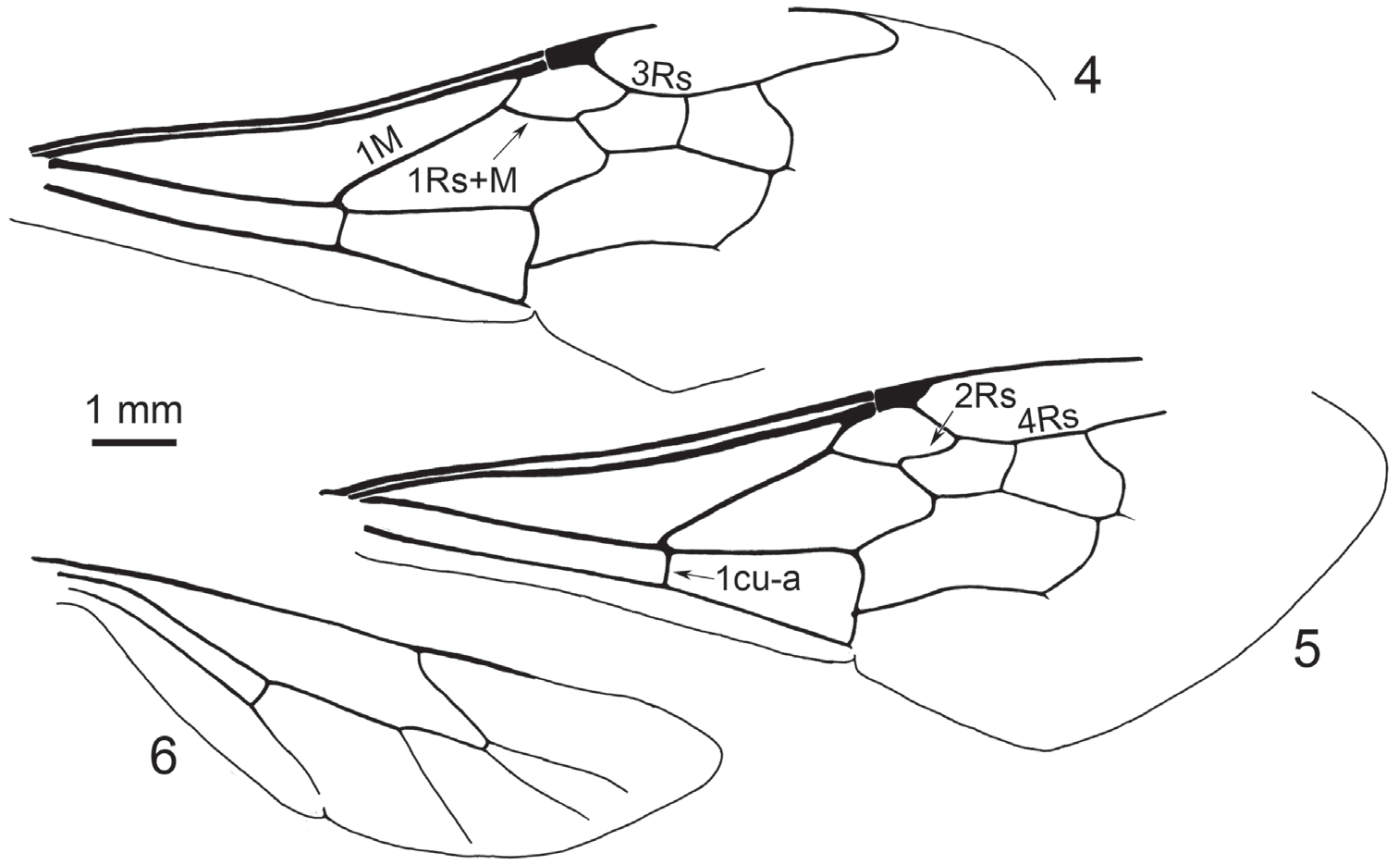
Line drawings of wing venation of holotype of Bombus (Cullumanobombus) trophonius, sp. n., as preserved. **4** Left forewing **5** Right forewing **6** Right hind wing.

**Specimen repository and descriptive terminology.** The fossil reported herein was retrieved from the collection of Zdeněk Dvořák, deposited in the museum holdings of the Bílina Mine Enterprises in Bílina, Czech Republic. The specimen was examined dry using a Nikon SMZ 645 stereomicroscope. Photographs were taken using a Canon EOS 550D digital camera coupled to a MP-E 65 mm macro lens. The description is provided here in the aim of improving diagnostic and species-level accounts of living and fossil bees (*e.g*., Engel 2011, Gonzalez et al. 2013). Morphological terminology follows that of Engel (2001a) and Michener (2007), with the format for the descriptions augmented from those of Wappler et al. (2012) and Dehon et al. (2014).

**Geometric morphometric analyses of forewing shape.** Prior to description using traditional venational traits, the present fossil was analyzed for its placement among other *Bombus* based on a geometric morphometric analysis of wing shape using vein landmarks. This method has proved useful in placing otherwise difficult to treat fossil species (*e.g*., Kotthoff et al. 2011, 2013, Dewulf et al. 2014, Dehon et al. 2017), including fossil bombines (Wappler et al. 2012, Dehon et al. in prep.). Geometric morphometric analysis (Pavlinov 2001) of insect wings is a valuable tool given that it is easily implemented, comparatively inexpensive, and the wings themselves are comparatively rigid, two-dimensional structures, species specific, and frequently well preserved in fossil specimens, albeit at times taphonomically distorted. Furthermore, forewing veins and their intersections are homologous among bees with three submarginal cells, like bumble bees (Ross 1936, Michener 2007). The method is rather robust at diagnosing and discriminating taxa at different levels (*e.g*., Pretorius 2005, Petit et al. 2006, Sadeghi et al. 2009, Francoy et al. 2012, Perrard et al. 2014), and has been employed successfully in palaeontological studies for evaluating the taxonomic affinities of otherwise difficult to determine fossils (*e.g*., Kennedy et al. 2009, Michez et al. 2009, Dehon et al. 2014, 2017, Dewulf et al. 2014, Perrard et al. 2016). Moreover, several studies have demonstrated the application of forewing shape analyses for discriminating subgenera, species, and populations of bumble bees (*e.g*., Aytekin et al. 2007, Wappler et al. 2012, Barkan and Aytekin 2013).

Morphometric analyses followed the procedures as outlined by Wappler et al. (2012) and Dehon et al. (2017, in prep.). As in Dehon et al. (2017), we employed three datasets to assess the taxonomic affinities of the fossil at different taxonomic levels by sampling broadly across extant and extinct tribes with the same number of submarginal cells as bumble bees. The first dataset consisted of a comprehensive sampling of bee tribes in order to maximize the shape diversity of our analyses, and this dataset was previously tested by Dehon et al. (2017). The dataset includes 20 specimens and four species per tribe, and whenever possible five specimens per species, and ultimately represented 979 female specimens from seven families, 18 subfamilies, 50 tribes, 135 genera, and 226 species. This first dataset was used to estimate the similarity of the fossil relative to the tribe Bombini (Suppl. material 1), and to determine a group of five tribes (i.e., Ancylaini, Tarsaliini, Emphorini, Euglossini, and Tetrapediini) exhibiting an overall similar wing shape to bumble bees. A second dataset sampled species more extensively across the tribe Bombini and the aforementioned four tribes with similar wing shapes. This was done in order to extend the shape diversity inside the target group. This dataset sampled 15 subgenera and 210 species of bumble bees, accounting for a total of 841 specimens, each species represented by a maximum of five specimens (Suppl. material 2). The dataset represented 100% of the subgeneric diversity and more than 80% of the world’s species. In addition, this second dataset included additional Ancylaini and Tarsaliini (two genera, nine species, and 25 specimens), Emphorini (four genera, 12 species, and 28 specimens), Euglossini (five genera, 11 species, and 55 specimens), and Tetrapediini (two genera, seven species, and 26 specimens) in the second dataset. Lastly, after confirmation of the affinities of the fossil with contemporary Bombini based on the second dataset, we considered a third dataset restricted entirely to bumble bee specimens so as to better assess the affinities of the fossil among modern subgenera of *Bombus* (*i.e*., the dataset from Suppl. material 2 with all groups except Bombini excluded).

For the reference datasets, left forewings were photographed using an Olympus SZH10 microscope combined with a Nikon D200 camera. Photographs were input in the software tps-UTIL 1.69 (Rohlf 2013a). The forewing shape was then captured by digitizing two-dimensional Cartesian coordinates of 18 landmarks on the wing venation and cells (refer to diagram of landmarks presented in Dehon et al., 2017: their figure 1), with the software tps-DIG version 2.27 (Rohlf 2013b). The configurations of the landmarks were superimposed using the GLS Procrustes superimposition in R version 3.0.2 (Rohlf and Slice 1990, Bookstein 1991, Adams and Otárola-Castillo 2013, R Development Core Team 2013). The closeness of the tangent space to the curved shape space was assessed using the software tps-SMALL v1.25 (Rohlf 2013c) by calculating the least-squares regression slope and the correlation coefficient between the Euclidean distances in the tangent space with the Procrustes distances in the shape space (Rohlf 1999). Prior to assignment of the Bílina fossil, discrimination of the wing shapes of the various taxa was assessed by Linear Discriminant Analyses (LDA) of the projected aligned landmark configurations. We did a LDA with the second dataset (*i.e*., bumblebees + five similar tribes), with tribe level as *a priori* groupings (Suppl. material 3) (a similar test was already performed for the first dataset by Dehon et al. (2017)). Lastly, we performed a LDA on the third dataset considering the subgenera as *a priori* groupings (Suppl. material 4).

**Figure 7.**
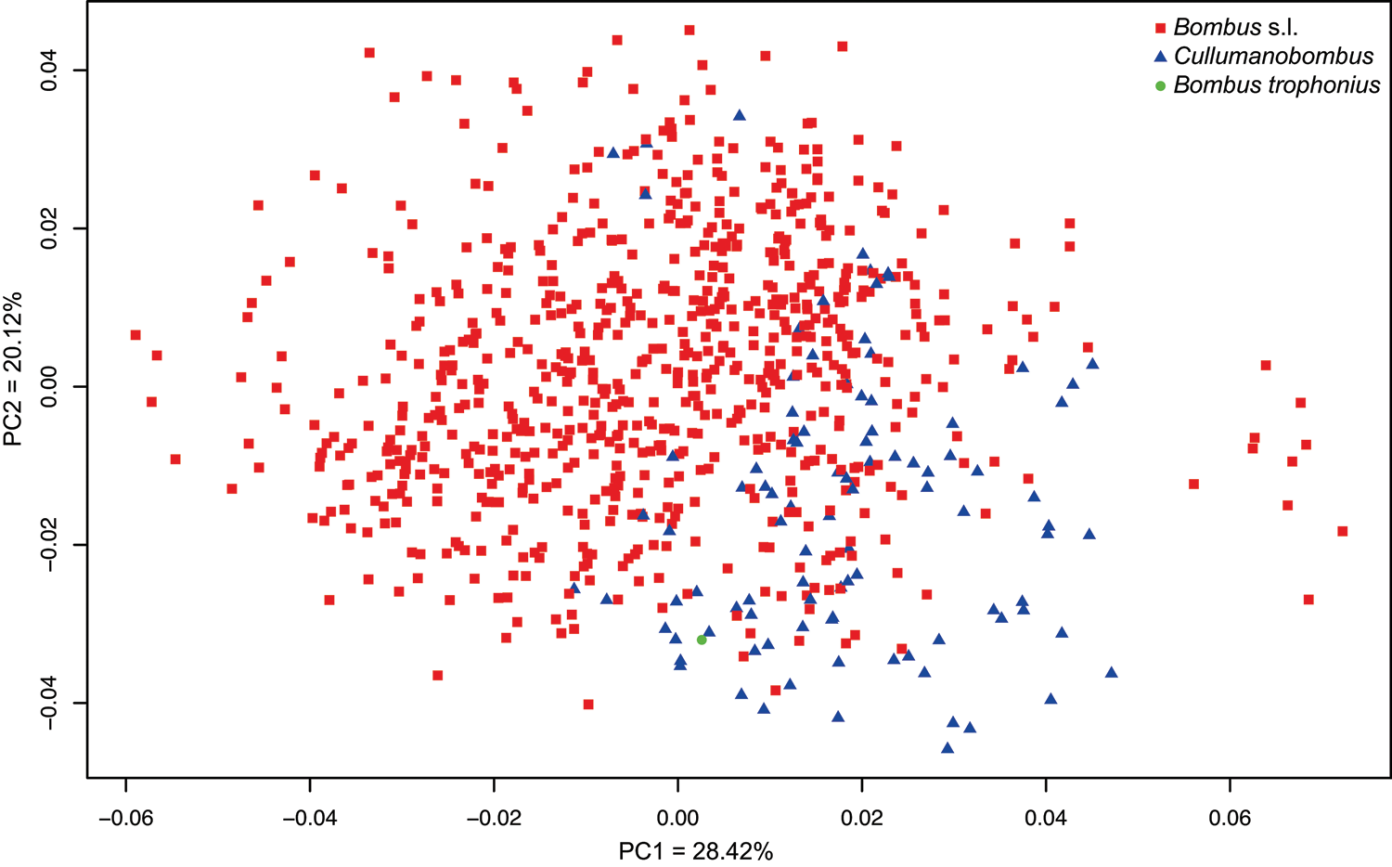
Ordination of the fossil along the two axes of the PCA (PC1 = 28.42% and PC2 = 20.12%) in the *Bombus* s.l. dataset, with extant specimens of *Cullumanobombus* highlighted in blue.

Discriminant analyses were performed by using the software R (R Development Core Team 2013). LDA effectiveness was assessed by the percentages of individuals correctly classified to their original taxon (*i.e*., hit-ratio) in a leave-one-out (LOO) cross-validation procedure based on the posterior probabilities (pp) of assignment. Given the observed scores of an “unknown”, the posterior probability equals the probability of the unit to belong to one group compared to all others. The unit is consequently assigned to the group for which the posterior probability is the highest (Huberty and Olejnik 2006). Taxonomic affinities of the Bílina fossil were assessed based on the score in the predictive discriminant space of shapes. Aligned coordinates of the specimens from the three datasets (including the fossil) were used to calculate the same five LDA as discussed above (*vide supra*). We included *a posteriori* the fossil in the five computed LDA space as an “unknown” specimen and calculated its score. Assignment of the fossil was estimated by calculating the Mahalanobis Distance (MD) between “unknown” and the group mean for each taxon (Suppl. materials 5–7). Principal Component Analyses (PCA) were also computed to visualize shape affinities between the fossil and the extant groups in the last dataset (Fig. 7).

## Results

**Shape variation within the datasets.** Analyses based on the first dataset with family, subfamily, and tribe *a priori* groupings are detailed in Dehon et al. (2017), with contemporary families, subfamilies, and tribes well discriminated. Contemporary tribes are also well discriminated in the second dataset (*i.e*., *Bombus* s.l. and most similar tribes), with a global hit-ratio of 99.6% (Suppl. material 3). Only the extinct tribes Electrapini and Melikertini are not well discriminated, with hit-ratios of 50.0% and 66.7%, respectively. Contemporary subgenera of *Bombus* s.l. are well discriminated in the bumble bee dataset, with a global hit-ratio of 87.4% and 106 misclassified specimens out of 841. Three subgenera show a hit-ratio of 100%: *Alpinobombus* Skorikov, *Kallobombus* Dalla Torre, and *Mendacibombus* Skorikov. Two subgenera have a hit-ratio between 90.0% and 99.0% – *Cullumanobombus* and *Psithyrus* Lepeletier – while two are poorly discriminated in the LDA – *Melanobombus* Dalla Torre and *Orientalibombus* Richards (72.1% and 70.0%, respectively) (Suppl. material 4). Overall, the results show a great reliability for classifying specimens based on the similarity of their forewing shape relative to our reference dataset of forewings. The cross-validation therefore allows us to be confident in the discrimination.

***A posteriori* assignment of the fossil.** The present fossil was assigned to Apidae, to “Non-parasitic Apidae”, and to Bombini by using the first dataset (Suppl. materials 5–7). When using the second dataset the fossil was assigned within *Bombus* s.l. (Suppl. material 8), and to subgenus Cullumanobombus by the third dataset (Suppl. material 9) (Fig. 7), although it could not discriminate the species as being part of the stem versus crown group. Accordingly, placement of the fossil from the Bílina Mine within *Cullumanobombus* is strongly supported by forewing shape. Continued work including all known fossil Bombini with living relatives will hopefully further refine this placement (Dehon et al. in prep.), particularly in combination with a heuristic phylogenetic exploration of forewing shape (analogous to that of Dehon et al. 2017).

### Systematic paleontology

#### Genus *Bombus* Latreille, 1802

##### Subgenus Cullumanobombus Vogt, 1911

###### Bombus (Cullumanobombus) trophonius sp. n.

Taxon classificationAnimaliaHymenopteraApidae

http://zoobank.org/9FBA6F95-5C97-4F9E-ABC0-EAA8F73403B7

Figs 1 , 2–3 , 4–6

Bombus sp. indet.; Prokop and Nel 2003: 166, Dvořák et al. 2010: 36, 78.

####### Diagnosis.

The new species has a wing shape that is consistent with species of the subgenus Cullumanobombus (Dehon et al. in prep.). Within this group, the fossil has a wing pattern most similar to Bombus (Cullumanobombus) rufocinctus Cresson, a species distributed widely across the Nearctic (Milliron 1973, Williams et al. 2014), with both species having a similar combination of 3Rs about as long as r-rs but shorter than 4Rs, the basal vein basad 1cu-a, 2Rs arched posteriorly but not as greatly prolonged proximally as in several other species of *Cullumanobombus* (*e.g*., Milliron 1971), 1m-cu entering second submarginal cell near midpoint (refer to Discussion). The convex pterostigmal border within the marginal cell, less apically narrowed marginal cell, and less arched 2rs-m minimally serve to distinguish the fossil species from *B. rufocinctus*.

####### Description.

♀: Wings and integument black as preserved (taphonomically altered; coloration and membrane pigmentation as in life unknown) (Figs 1–3); forewing total length 14.6 mm; maximum width 5.10 mm (Figs 2, 4, 5); basal vein (1M) weakly arched at base, comparatively straight along length, basad 1cu-a by about vein width, in line with 1Rs; Rs+M originating anteriorad, 1Rs slightly shorter than r-rs; pterostigma short, slightly longer than wide, border inside marginal cell convex, prestigma nearly as long as pterostigma; marginal cell length 5.1 mm, width 1.1 mm, tapering slightly across its length, free portion of cell slightly shorter than portion bordering submarginal cells, apex rounded and offset from anterior wing margin by much more than vein width, not appendiculate; 2Rs strongly arched basally and then gently arched outward, giving second submarginal cell distinct proximal extension; r-rs about as long as 3Rs; 4Rs only slightly longer than 3Rs; three submarginal cells of comparatively similar sizes, albeit third slightly larger than first or second; first submarginal cell length 0.9 mm, width 1.0 mm; second submarginal cell length 1.3 mm, width 0.9 mm; third submarginal cell length 1.6 mm, width 1.2 mm; 1rs-m straight, comparatively orthogonal with Rs; 2rs-m arched distally in posterior half; 1m-cu distinctly angulate anteriorly near M, entering second submarginal cell near cell’s midlength; 2m-cu weakly and gently arched apically, meeting third submarginal cell at cell’s apical fifth of length. Hind wing length 9.4 mm, width 2.6 mm (Figs 3, 6). Preserved portion of thorax and legs difficult to discern and interpret, although portion of metatibial corbicula preserved (basal quarter to third), and most sclerites with numerous, long setae.

♂: *Latet*.

####### Holotype.

♀ (caste uncertain, likely a worker), ZD0003, Early Miocene, Most Formation, Clayey Superseam Horizon, Holešice Member (No. 30), Bílina Mine near Bílina, Czech Republic; deposited in the museum collection of the Bílina Mine Enterprises, Bílina, Czech Republic.

####### Etymology.

The specific epithet is taken from the Greek mythological hero, Trophonius, one of the two brothers who absconded with the treasure of King Hyrieus and who fled into caverns at Lebadaea (today’s Livadeia in Boeotia). Trophonius is generally associated with bees and the underworld since, according to legend, it was a swarm of bees that led a boy to rediscover his cave, bringing his spirit honor and peace.

## Discussion

Naturally, it is challenging in the absence of clear characters from the head, mandibles, genitalia, or patterns of coloration to make a globally satisfactory assessment of the present fossil. Nonetheless, a morphometric shape analysis of the fossil among other living and fossil bombines confidently placed *B. trophonius* within the subgenus Cullumanobombus, in the broad sense as advocated by Williams et al. (2008). Most species of *Cullumanobombus* have New World distributions, except for *B. cullumanus* (Kirby), *B. semenoviellus* Skorikov, *B. unicus* Morawitz, and *B. vogti* Friese which are found in the Old World (Milliron 1973, Williams 1985). The overall combination of wing traits tends to exclude *B. trophonius* from all groups within *Cullumanobombus* with the exception of one. For example, in most species of the *robustus*, *fraternus*, *griseocollis*, *cullumanus*, *rubicundus*, and *brachycephalus* species groups 3Rs is longer than r-rs (rather than about as long as r-rs in *B. trophonius*), and in some, such as the latter two groups, it is also longer than 4Rs (rather than 4Rs longer than 3Rs as is the case in *B. trophonius*). In addition, in several groups 1m-cu enters the second submarginal cell basal its midpoint (*e.g*., *brachycephalus* and *fraternus* groups), rather than near the midpoint in *B. trophonius*. The second submarginal cell is frequently more pronouncedly elongate proximally, owing to a more dramatically arched 2Rs, in many species of the *rubicundus* and *robustus* groups, while 2rs-m is less arched in the *brachycephalus*, *robustus*, *fraternus*, and *griseocollis* groups and the basal vein and 1cu-a are usually confluent in B. (C.) brachycephalus Handlirsch. The only species within the clade that has the same combination of features as are present in the fossil is B. (C.) rufocinctus. The latter species is common from North Amercia to Mexico. Interestingly, *B. rufocinctus* is considered basal within *Cullumanobombus* (Cameron et al. 2007, Hines 2008), and the overall shared pattern between their wings may be symplesiomorphies (based on the plesiomorphic placement of *B. rufocinctus* and its wing venation relative to more derived species of *Cullumanobombus*), which would be intuitively pleasing if *B. trophonius* were representative of a stem group to the subgenus. In *B. rufocinctus* the marginal cell is often more narrowed apically than in *B. trophonius*, and the former has worker forewing lengths shorter than in the fossil (approximately 11 mm in *B. rufocinctus*, versus over 14 mm in *B. trophonius*). However, queens of *B. rufocinctus* can easily exceed 14 mm in forewing length, and if the holotype of *B. trophonius* was a queen, then the two would be of approximately similar proportions. The age of *B. trophonius* is in general accordance with what one might except of a stem-group *Cullumanobombus* based on the divergence time estimations of Hines (2008). The palaeoclimate of the Bílina locality was subtropical/warm temperate and temperate (Kvaček et al. 2004), while extant species of *Cullumanobombus* exploit a wide variety of climatic niches, mainly dry and warm, but not boreal. While there remains a plethora of questions regarding the complete characterization of *B. trophonius*, the species apparently represents an important record for *Cullumanobombus* and the discovery of more complete material in the future will undoubtedly continue to bring revelations regarding bumble bee evolution and biogeography during the Neogene.

## Supplementary Material

XML Treatment for Bombus (Cullumanobombus) trophonius

## Supplementary materials

Supplementary material 1 Table S1 Data type: Microsoft Word Document (.docx) Explanation note: First dataset for geometric morphometric analyses encompassing 988 specimens from 234 species, 141 genera, 53 tribes, 18 subfamilies, and seven families of Anthophila (Apoidea). All included groups have three submarginal cells. N1= number of species; N2 = number of specimens. This dataset is made available under the Open Database License (http://opendatacommons.org/licenses/odbl/1.0/). The Open Database License (ODbL) is a license agreement intended to allow users to freely share, modify, and use this Dataset while maintaining this same freedom for others, provided that the original source and author(s) are credited.Jakub Prokop, Manuel Dehon, Denis Michez, Michael S. Engel

Supplementary material 2 Table S2 Data type: Microsoft Excel Worksheet (.xlsx) Explanation note: Second dataset for the geometric morphometric analyses. This sample includes 872 specimens from 247 species, 14 genera, and six tribes of Apidae. N = number of specimens. This dataset is made available under the Open Database License (http://opendatacommons.org/licenses/odbl/1.0/). The Open Database License (ODbL) is a license agreement intended to allow users to freely share, modify, and use this Dataset while maintaining this same freedom for others, provided that the original source and author(s) are credited.Jakub Prokop, Manuel Dehon, Denis Michez, Michael S. Engel

Supplementary material 3 Table S3 Data type: Microsoft Excel Worksheet (.xlsx) Explanation note: Specimen assignment in tribes using the cross-validation procedure in the LDA of forewing shape in the “*Bombus* s.l. + comparison groups” dataset. Original groups are along the rows, predicted groups are along the columns. The hit ratio (HR%) is given for each tribe. This dataset is made available under the Open Database License (http://opendatacommons.org/licenses/odbl/1.0/). The Open Database License (ODbL) is a license agreement intended to allow users to freely share, modify, and use this Dataset while maintaining this same freedom for others, provided that the original source and author(s) are credited.Jakub Prokop, Manuel Dehon, Denis Michez, Michael S. Engel

Supplementary material 4 Table S4 Data type: Microsoft Excel Worksheet (.xlsx) Explanation note: Specimen assignment in subgenera using the cross-validation procedure in the LDA of forewing shape in the dataset of *Bombus* s.l. Original groups are along the rows, predicted groups are along the columns. The hit ratio (HR%) is given for each subgenus. This dataset is made available under the Open Database License (http://opendatacommons.org/licenses/odbl/1.0/). The Open Database License (ODbL) is a license agreement intended to allow users to freely share, modify, and use this Dataset while maintaining this same freedom for others, provided that the original source and author(s) are credited.Jakub Prokop, Manuel Dehon, Denis Michez, Michael S. Engel

Supplementary material 5 Table S5 Data type: Microsoft Excel Worksheet (.xlsx) Explanation note: Mahalanobis distances (MD) between familial centroids and the 979 specimens, and the fossils and familial centroids in the tribal dataset. This dataset is made available under the Open Database License (http://opendatacommons.org/licenses/odbl/1.0/). The Open Database License (ODbL) is a license agreement intended to allow users to freely share, modify, and use this Dataset while maintaining this same freedom for others, provided that the original source and author(s) are credited.Jakub Prokop, Manuel Dehon, Denis Michez, Michael S. Engel

Supplementary material 6 Table S6 Data type: Microsoft Excel Worksheet (.xlsx) Explanation note: Mahalanobis distances (MD) between subfamilial centroids and the 979 specimens, and the fossils and subfamilial centroids in the tribal dataset. This dataset is made available under the Open Database License (http://opendatacommons.org/licenses/odbl/1.0/). The Open Database License (ODbL) is a license agreement intended to allow users to freely share, modify, and use this Dataset while maintaining this same freedom for others, provided that the original source and author(s) are credited.Jakub Prokop, Manuel Dehon, Denis Michez, Michael S. Engel

Supplementary material 7 Table S7 Data type: Microsoft Excel Worksheet (.xlsx) Explanation note: Mahalanobis distances (MD) between tribal centroids and the 979 specimens, and the fossils and tribal centroids in the tribal dataset. This dataset is made available under the Open Database License (http://opendatacommons.org/licenses/odbl/1.0/). The Open Database License (ODbL) is a license agreement intended to allow users to freely share, modify, and use this Dataset while maintaining this same freedom for others, provided that the original source and author(s) are credited.Jakub Prokop, Manuel Dehon, Denis Michez, Michael S. Engel

Supplementary material 8 Table S8 Data type: Microsoft Excel Worksheet (.xlsx) Explanation note: Mahalanobis distances (MD) between tribal centroids and the 975 specimens, and the fossils and tribal centroids in the “*Bombus* s.l. + comparison groups” dataset. This dataset is made available under the Open Database License (http://opendatacommons.org/licenses/odbl/1.0/). The Open Database License (ODbL) is a license agreement intended to allow users to freely share, modify, and use this Dataset while maintaining this same freedom for others, provided that the original source and author(s) are credited.Jakub Prokop, Manuel Dehon, Denis Michez, Michael S. Engel

Supplementary material 9 Table S9 Data type: Microsoft Excel Worksheet (.xlsx) Explanation note: Mahalanobis distances (MD) between subgeneric centroids and the 841 specimens, and the fossils and subgeneric centroids in the *Bombus* s.l. dataset. This dataset is made available under the Open Database License (http://opendatacommons.org/licenses/odbl/1.0/). The Open Database License (ODbL) is a license agreement intended to allow users to freely share, modify, and use this Dataset while maintaining this same freedom for others, provided that the original source and author(s) are credited.Jakub Prokop, Manuel Dehon, Denis Michez, Michael S. Engel

## References

[B1] Adams DC Otárola-Castillo E (2013) Geomorph: an R package for the collection and analysis of geometric morphometric shape data. Methods in Ecology and Evolution 4(4): 393–399. https://doi.org/10.1111/2041-210X.12035

[B2] Aytekin AM Terzo M Rasmont P Çağatay N (2007) Landmark based geometric morphometric analysis of wing shape in *Sibiricobombus* Vogt (Hymenoptera: Apidae: *Bombus* Latreille). Annales de la Sociéte Entomologique de France 43(1): 95–102. https://doi.org/10.1080/00379271.2007.10697499

[B3] Barkan NP Aytekin AM (2013) Systematical studies on the species of the subgenus Bombus (Thoracobombus) (Hymenoptera: Apidae, *Bombus* Latreille) in Turkey. Zootaxa 3737(2): 167–183. https://doi.org/10.11646/zootaxa.3737.2.5 25112746 10.11646/zootaxa.3737.2.5

[B4] Bookstein FL (1991) Morphometric tools for landmark data: geometry and biology. Cambridge University Press, Cambridge, 435 pp.

[B5] Camargo JMF Grimaldi DA Pedro SRM (2000) The extinct fauna of stingless bees (Hymenoptera: Apidae: Meliponini) in Dominican amber: Two new species and redescription of the male of *Proplebeia dominicana* (Wille and Chandler). American Museum Novitates 3293: 1–24. https://doi.org/10.1206/0003-0082(2000)293<0001:TEFOSB>2.0.CO;2

[B6] Cameron SA Hines HM Williams PH (2007) A comprehensive phylogeny of the bumble bees (*Bombus*). Biological Journal of the Linnean Society 91(1): 161–188.

[B7] Cardinal S Packer L (2007) Phylogenetic analysis of the corbiculate Apinae based on morphology of the sting apparatus (Hymenoptera: Apidae). Cladistics 23(2): 99–118. https://doi.org/10.1111/j.1095-8312.2007.00784.x 10.1111/j.1096-0031.2006.00137.x 34905847

[B8] Cockerell TDA (1908) Descriptions and records of bees–XX. Annals and Magazine of Natural History, Eighth Series 2(10): 323–334.

[B9] Dehon M Michez D Nel A Engel MS De Meulemeester T (2014) Wing shape of four new bee fossils (Hymenoptera: Anthophila) provides insights to bee evolution. PLOS ONE 9(10): e108865. https://doi.org/10.1371/journal.pone.0108865 10.1371/journal.pone.0108865 PMC4212905 25354170

[B10] Dehon M Perrard A Engel MS Nel A Michez D (2017) Antiquity of cleptoparasitism among bees revealed by morphometric and phylogenetic analysis of a Paleocene fossil nomadine (Hymenoptera: Apidae). Systematic Entomology 42(3): 543–554. https://doi.org/10.1111/syen.12230

[B11] Dewulf A De Meulemeester T Dehon M Engel MS Michez D (2014) A new interpretation of the bee fossil *Melitta willardi* Cockerell (Hymenoptera, Melittidae) based on geometric morphometrics of the wing. ZooKeys 389: 35–48. https://doi.org/10.3897/zookeys.389.7076 10.3897/zookeys.389.7076 PMC3974431 24715773

[B12] Dvořák Z Mach K Prokop J Knor S (2010) Třetihorní Fauna Severočeské Hnědouhelné Pánve. Nakladatelství Granit, Prague, 175 pp.

[B13] Engel MS (1998a) A new species of the Baltic amber bee genus *Electrapis* (Hymenoptera: Apidae). Journal of Hymenoptera Research 7(1): 94–101.

[B14] Engel MS (1998b) Fossil honey bees and evolution in the genus *Apis* (Hymenoptera: Apidae). Apidologie 29(3): 265–281. https://doi.org/10.1051/apido:19980306

[B15] Engel MS (1999a) The taxonomy of Recent and fossil honey bees (Hymenoptera: Apidae; *Apis*). Journal of Hymenoptera Research 8(2): 165–196.

[B16] Engel MS (1999b) The first fossil *Euglossa* and phylogeny of the orchid bees (Hymenoptera: Apidae; Euglossini). American Museum Novitates 3272: 1–14.

[B17] Engel MS (2000a) Fossils and phylogeny: A paleontological perspective on social bee evolution. In: Bitondi MMG Hartfelder K (Eds) Anais do IV Encontro sobre Abelhas. Universidade de São Paulo, Ribeirão Preto, 217–224.

[B18] Engel MS (2000b) A new interpretation of the oldest fossil bee (Hymenoptera: Apidae). American Museum Novitates 3296: 1–11. https://doi.org/10.1206/0003-0082(2000)3296<0001:ANIOTO>2.0.CO;2

[B19] Engel MS (2001a) A monograph of the Baltic amber bees and evolution of the Apoidea (Hymenoptera). Bulletin of the American Museum of Natural History 259: 1–192. https://doi.org/10.1206/0003-0090(2001)259<0001:AMOTBA>2.0.CO;2

[B20] Engel MS (2001b) Monophyly and extensive extinction of advanced eusocial bees: Insights from an unexpected Eocene diversity. Proceedings of the National Academy of Sciences, USA 98(4): 1661–1664. https://doi.org/10.1073/pnas.98.4.1661 10.1073/pnas.041600198 PMC29313 11172007

[B21] Engel MS (2006) A giant honey bee from the middle Miocene of Japan (Hymenoptera: Apidae). American Museum Novitates 3504: 1–12. https://doi.org/10.1206/0003-0082(2006)504[0001:AGHBFT]2.0.CO;2

[B22] Engel MS (2011) Systematic melittology: where to from here? Systematic Entomology 36(1): 2–15. https://doi.org/10.1111/j.1365-3113.2010.00544.x

[B23] Engel MS (2014) An orchid bee of the genus *Eulaema* in Early Miocene Mexican amber (Hymenoptera: Apidae). Novitates Paleoentomologicae 7: 1–15. https://doi.org/10.17161/np.v0i7.4726

[B24] Engel MS Michener CD (2013a) A minute stingless bee in Eocene Fushan [sic] amber from northeastern China (Hymenoptera: Apidae). Journal of Melittology 14: 1–10.

[B25] Engel MS Michener CD (2013b) Geological history of the stingless bees (Apidae: Meliponini). In: Vit P Roubik DW (Eds) Stingless Bees Process Honey and Pollen in Cerumen Pots. Facultad de Farmacia y Bioanálisis, Universidad de Los Andes, Mérida, 1–7.

[B26] Engel MS Hinojosa-Díaz IA Rasnitsyn AP (2009) A honey bee from the Miocene of Nevada and the biogeography of *Apis* (Hymenoptera: Apidae: Apini). Proceedings of the California Academy of Sciences, Series 4 60(3): 23–38.

[B27] Engel MS Ortega-Blanco J Nascimbene PC Singh H (2013) The bees of Early Eocene Cambay amber (Hymenoptera: Apidae). Journal of Melittology 25: 1–12. https://doi.org/10.17161/jom.v0i25.4659

[B28] Engel MS Breitkreuz LCV Ohl M (2014) The first male of the extinct bee tribe Melikertini (Hymenoptera: Apidae). Journal of Melittology 30: 1–18. https://doi.org/10.17161/jom.v0i30.4698

[B29] Fikáček M Hájek J Prokop J (2008) New records of the water beetles (Coleoptera: Dytiscidae, Hydrophilidae) from the central European Oligocene-Miocene deposits, with a confirmation of the generic attribution of *Hydrobiomorpha enspelense* Wedmann 2000. Annales de la Société Entomologique de France 44(2): 187–199. https://doi.org/10.1080/00379271.2008.10697555

[B30] Francoy TM de Faria Franco F Roubik DW (2012) Integrated landmark and outline-based morphometric methods efficiently distinguish species of *Euglossa* (Hymenoptera, Apidae, Euglossini). Apidologie 43(6): 609–617. https://doi.org/10.1007/s13592-012-0132-2

[B31] Gonzalez VH Griswold T Engel MS (2013) Obtaining a better taxonomic understanding of native bees: where do we start? Systematic Entomology 38(4): 645–653. https://doi.org/10.1111/syen.12029

[B32] Greco MK Welz PM Siegrist M Ferguson SJ Gallmann P Roubik DW Engel MS (2011) Description of an ancient social bee trapped in amber using diagnostic radioentomology. Insectes Sociaux 58(4): 487–494. https://doi.org/10.1007/s00040-011-0168-8

[B33] Grímsson F Zetter R Labandeira CC Engel MS Wappler T (2017) Taxonomic description of *in situ* bee pollen from the middle Eocene of Germany. Grana 56(1): 37–70. https://doi.org/10.1080/00173134.2015.1108997 28057943 10.1080/00173134.2015.1108997 PMC5161302

[B34] Hansen J Sato M Russell G Kharecha P (2013) Climate sensitivity, sea level and atmospheric carbon dioxide. Philosophical Transactions of the Royal Society A 371(2001): 20120294. https://doi.org/10.1098/rsta.2012.0294 10.1098/rsta.2012.0294 PMC3785813 24043864

[B35] Hines HM (2008) Historical biogeography, divergence times, and diversification patterns of bumble bees (Hymenoptera: Apidae: *Bombus*). Systematic Biology 57(1): 58–75. https://doi.org/10.1080/10635150801898912 18275002 10.1080/10635150801898912

[B36] Hinojosa-Díaz IA Engel MS (2007) A new fossil orchid bee in Colombian copal (Hymenoptera: Apidae). American Museum Novitates 3589: 1–7. https://doi.org/10.1206/0003-0082(2007)3589[1:ANFOBI]2.0.CO;2

[B37] Huberty CJ Olejnik S (2006) Applied MANOVA and Discriminant Analysis [2^nd^ Edition]. Wiley, New Jersey, 488 pp https://doi.org/10.1002/047178947X

[B38] Kawakita A Ascher JS Sota T Kato M Roubik DW (2008) Phylogenetic analysis of the corbiculate bee tribes based on 12 nuclear protein-coding genes (Hymenoptera: Apoidea: Apidae). Apidologie 39(1): 163–175. https://doi.org/10.1051/apido:2007046

[B39] Kennedy WJ Reyment RA MacLeod N Krieger J (2009) Species discrimination in the Lower Cretaceous (Albian) ammonite genus *Knemiceras* von Buch, 1848. Palaeontographica, Abteilung A: Paläozoologie–Stratigraphie 290(1–3): 1–63. https://doi.org/10.1127/pala/290/2009/1

[B40] Knor S Prokop J Kvaček Z Janovský Z Wappler T (2012) Plant-arthropod associations from the Early Miocene of the Most Basin in North Bohemia – palaeoecological and palaeoclimatological implications. Palaeogeography, Palaeoclimatology, Palaeoecology 321–322: 102–112. https://doi.org/10.1016/j.palaeo.2012.01.023

[B41] Knor S Skuhravá M Wappler T Prokop J (2013) Galls and gall makers on plant leaves from the Lower Miocene (Burdigalian) of the Czech Republic: systematic and palaeoecological implications. Review of Palaeobotany and Palynology 188: 38–51. https://doi.org/10.1016/j.revpalbo.2012.10.001

[B42] Kotthoff U Wappler T Engel MS (2011) Miocene honey bees from the Randeck Maar, southwestern Germany (Hymenoptera, Apidae). ZooKeys 96: 11–37. https://doi.org/10.3897/zookeys.96.752 10.3897/zookeys.96.752 PMC3095134 21594072

[B43] Kotthoff U Wappler T Engel MS (2013) Greater past disparity and diversity hints at ancient migrations of European honey bee lineages into Africa and Asia. Journal of Biogeography 40(10): 1832–1838. https://doi.org/10.1111/jbi.12151

[B44] Kvaček Z Böhme M Dvořák Z Konzalová M Mach K Prokop J Rajchl M (2004) Early Miocene freshwater and swamp ecosystems of the Most Basin (northern Bohemia) with particular reference to the Bílina Mine section. Journal of the Czech Geological Society 49(1–2): 1–40.

[B45] Kwong WK Medina LA Koch H Sing K-W Soh EJY Ascher JS Jaffé R Moran NA (2017) Dynamic microbiome evolution in social bees. Science Advances 3(3): e1600513. https://doi.org/10.1126/sciadv.1600513 10.1126/sciadv.1600513 PMC5371421 28435856

[B46] Latreille PA (1802) Histoire naturelle des fourmis, et recueil de memoires et d’observations sur les abeilles, les araignées, les faucheurs, et autres insectes. Crapelet, Paris, 445 pp.

[B47] Michener CD (1982) A new interpretation of fossil social bees from the Dominican Republic. Sociobiology 7(1): 37–45.

[B48] Michener CD (1990) Classification of the Apidae (Hymenoptera). University of Kansas Science Bulletin 54(4): 75–163.

[B49] Michener CD (2007) The Bees of the World [2^nd^ Edition]. Johns Hopkins University Press, Baltimore, 953 pp. [20 pls.]

[B50] Michener CD Grimaldi DA (1988) A *Trigona* from Late Cretaceous amber of New Jersey (Hymenoptera: Apidae: Meliponinae). American Museum Novitates 2917: 1–10.

[B51] Michez D De Meulemeester T Nel A Rasmont P Patiny S (2009) New fossil evidence of the early diversification of bees: *Paleohabropoda oudardi* from the French Paleocene (Hymenoptera, Apidae, Anthophorini). Zoologica Scripta 38(2): 171–181 https://doi.org/10.1111/j.1463-6409.2008.00362.x

[B52] Michez D Vanderplanck M Engel MS (2012) Fossil bees and their plant associates. In: Patiny S (Ed.) Evolution of Plant-Pollinator Relationships. Cambridge University Press, Cambridge, 103–164.

[B53] Milliron HE (1971) A monograph of the Western Hemisphere bumblebees (Hymenoptera: Apidae; Bombinae). I. The genera *Bombus* and Megabombus subgenus Bombias. Memoirs of the Entomological Society of Canada 82: 1–80. https://doi.org/10.4039/entm10382fv

[B54] Milliron HE (1973) A monograph of the Western Hemisphere bumblebees (Hymenoptera: Apidae; Bombinae). III. The genus Pyrobombus subgenus Cullumanobombus. Memoirs of the Entomological Society of Canada 91: 239–333.

[B55] Noll FB (2002) Behavioral phylogeny of corbiculate Apidae (Hymenoptera; Apinae), with special reference to social behavior. Cladistics 18(2): 137–153. https://doi.org/10.1111/j.1096-0031.2002.tb00146.x 10.1111/j.1096-0031.2002.tb00146.x 34911221

[B56] Patiny S Engel MS Vanmarsenille P Michez D (2007) A new record of *Thaumastobombus andreniformis* Engel 2001 in Eocene amber (Hymenoptera: Apidae). Annales de la Société Entomologique de France 43(4): 505–508. https://doi.org/10.1080/00379271.2007.10697540

[B57] Pavlinov IY (2001) Geometric morphometrics, a new analytical approach to comparison of digitized images. Information Technology in Biodiversity Research, Abstracts of the 2^nd^ International Symposium, Saint Petersburg, 41–90.

[B58] Perrard A Baylac M Carpenter JM Villemant C (2014) Evolution of wing shape in hornets: why is the wing venation efficient for species identification? Journal of Evolutionary Biology 27(12): 2665–2675. https://doi.org/10.1111/jeb.12523 10.1111/jeb.12523 25345804

[B59] Perrard A Lopez-Osorio F Carpenter JM (2016) Phylogeny, landmark analysis and the use of wing venation to study the evolution of social wasps (Hymenoptera: Vespidae: Vespinae). Cladistics 32(4): 406–425. https://doi.org/10.1111/cla.12138 10.1111/cla.12138 34740304

[B60] Pešek J Brož B Brzobohatý R Dašková J Doláková N Elznic A Fejfar O Franců J Hladilová Š Holcová K Honěk J Hoňková K Kvaček J Kvaček Z Macůrek V Mikuláš R Opluštil S Rojík P Spudil J Svobodová M Sýkorová I Švábenická L Teodoridis V Tomanová-Petrová P (2014) Tertiary Basins and Lignite Deposits of the Czech Republic. Czech Geological Survey, Prague, 284 pp.

[B61] Petit D Picaud F Elghadraoui L (2006) Géométrie morphologique des ailes des Acrididae (Orthoptera: Caelifera): sexe, stridulation, caractère. Annales de la Société Entomologique de France 42(1): 63–73. https://doi.org/10.1080/00379271.2006.10697450

[B62] Pound MJ Salzmann U (2017) Heterogeneity in global vegetation and terrestrial climate change during the Late Eocene to Early Oligocene transition. Scientific Reports 7: 43386. https://doi.org/10.1038/srep43386 10.1038/srep43386 PMC5324063 28233862

[B63] Pretorius E (2005) Using geometric morphometrics to investigate wing dimorphism in males and females of Hymenoptera – a case study based on the genus *Tachysphex* Kohl (Hymenoptera: Sphecidae: Larrinae). Australian Journal of Entomology 44(2): 113–121. https://doi.org/10.1111/j.1440-6055.2005.00464.x

[B64] Prokop J (2003) Remarks on palaeoenvironmental changes based on reviewed Tertiary insect associations from the Krušné hory piedmont basins and the České Středohoří Mts in northwestern Bohemia (Czech Republic). Acta Zoologica Cracoviensia 46(Supplement-Fossil Insects): 329–344.

[B65] Prokop J Nel A (2000) *Merlax bohemicus* gen. n., sp. n., a new fossil dragonfly from the Lower Miocene of northern Bohemia (Odonata: Aeshnidae). European Journal of Entomology 97(3): 427–431. https://doi.org/10.14411/eje.2000.065

[B66] Prokop J Nel A (2003) New fossil Aculeata from the Oligocene of the České Středohoří Mts and the Lower Miocene of the Most Basin in northern Czech Republic (Hymenoptera: Apidae, Vespidae). Acta Musei Nationalis Pragae, Series B, Natural History 59(3–4): 163–171 [1 pl.]

[B67] Prokop J Wappler T Knor S Kvaček Z (2010) Plant-arthropod associations from the Lower Miocene of the Most Basin in northern Bohemia (Czech Republic): a preliminary report. Acta Geologica Sinica 84(4): 903–914. https://doi.org/10.1111/j.1755-6724.2010.00262.x

[B68] R Development Core Team (2013) A language and environment for statistical computing, version 3.0.2, ISBN 3-900051-07-0, R Foundation for Statistical Computing, Vienna.

[B69] Rajchl M Uličný D Grygar R Mach K (2009) Evolution of basin architecture in an incipient continental rift: The Cenozoic Most Basin, Eger Graben (central Europe). Basin Research 21(3): 269–294. https://doi.org/10.1111/j.1365-2117.2008.00393.x

[B70] Rasmont P Franzén M Lecocq T Harpke A Roberts SPM Biesmeijer JC Castro L Cederberg B Dvořák L Fitzpatrick Ú Gonseth Y Haubruge E Mahé G Manino A Michez D Neumayer J Ødegaard F Paukkunen J Pawlikowski T Potts SG Reemer M Settele J Straka J Schweiger O (2015) Climatic risk and distribution atlas of European bumblebees. BioRisk 10: 1–236. https://doi.org/10.3897/biorisk.10.4749

[B71] Rohlf FJ (1999) Shape statistics: Procrustes superimpositions and tangent spaces. Journal of Classification 16(2): 197–223. https://doi.org/10.1007/s003579900054

[B72] Rohlf FJ (2013a) tpsUTIL Version 1.56. Department of Ecology and Evolution, State University of New York at Stony Brook, New York.

[B73] Rohlf FJ (2013b) tpsDIG Version 2.17. Department of Ecology and Evolution, State University of New York at Stony Brook, New York.

[B74] Rohlf FJ (2013c) tpsSMALL Version 1.25. Department of Ecology and Evolution, State University of New York at Stony Brook, New York.

[B75] Rohlf FJ Slice D (1990) Extensions of the Procrustes method for the optimal superimposition of landmarks. Systematic Zoology 39(1): 40–59. https://doi.org/10.2307/2992207

[B76] Ross HH (1936) The ancestry and wing venation of the Hymenoptera. Annals of the Entomological Society of America 29(1): 99–111. https://doi.org/10.1093/aesa/29.1.99

[B77] Sadeghi S Adriaens D Dumont HJ (2009) Geometric morphometric analysis of wing shape variation in ten European populations of *Calopteryx splendens* (Harris, 1782) (Zygoptera: Calopterygidae). Odonatologica 38(4): 341–357.

[B78] Schultz TR Engel MS Prentice M (1999) Resolving conflict between morphological and molecular evidence for the origin of eusociality in the corbiculate bees (Hymenoptera: Apidae): a hypothesis-testing approach. University of Kansas Natural History Museum Special Publication 24: 125–138.

[B79] Schultz TR Engel MS Ascher JS (2001) Evidence for the origin of eusociality in the corbiculate bees (Hymenoptera: Apidae). Journal of the Kansas Entomological Society 74(1): 10–16.

[B80] Shrbený O Bůžek Č Čtyroký P Fejfar O Konzalová M Kvaček Z Malecha A Šantrůček P Václ J (1994) Terciér Českého masívu [Tertiary of the Bohemian Massif]. In: Klomínský J (Ed.) Geologický Atlas České Republiky. Stratigrafie [Geological Atlas of the Czech Republic]. Český Geologický Ústav, Prague, map 3. [In Czech and English]

[B81] Vogt O (1911) Studien über das Artproblem. 2 Mitteilung. Über das Variieren der Hummeln. 2 Teil. Sitzungsberichte der Gesellschaft Natuforschender Freunde zu Berlin 1911: 31–74.

[B82] Wappler T Engel MS (2003) The middle Eocene bee faunas of Eckfeld and Messel, Germany (Hymenoptera, Apoidea). Journal of Paleontology 77(5): 908–921. https://doi.org/10.1017/S0022336000044760

[B83] Wappler T De Meulemeester T Aytekin AM Michez D Engel MS (2012) Geometric morphometric analysis of a new Miocene bumble bee from the Randeck Maar of southwestern Germany (Hymenoptera: Apidae). Systematic Entomology 37(4): 784–792. https://doi.org/10.1111/j.1365-3113.2012.00642.x

[B84] Wappler T Dlussky GM Engel MS Prokop J Knor S (2014) A new trap-jaw ant species of the genus *Odontomachus* (Hymenoptera: Formicidae: Ponerinae) from the Early Miocene (Burdigalian) of the Czech Republic. Paläontologische Zeitschrift 88(4): 495–502. https://doi.org/10.1007/s12542-013-0212-2

[B85] Wappler T Labandeira CC Engel MS Zetter R Grímsson F (2015) Specialized and generalized pollen-collection strategies in an ancient bee lineage. Current Biology 25(23): 3092–3098. https://doi.org/10.1016/j.cub.2015.09.021 26585282 10.1016/j.cub.2015.09.021 PMC6485464

[B86] Williams PH (1985) A preliminary cladistic investigation of relationships among the bumble bees (Hymenoptera, Apidae). Systematic Entomology 10(2): 239–255. https://doi.org/10.1111/j.1365-3113.1985.tb00529.x

[B87] Williams PH Cameron SA Hines HM Cederberg B Rasmont P (2008) A simplified subgeneric classification of the bumblebees (genus *Bombus*). Apidologie 39(1): 46–74. https://doi.org/10.1051/apido:2007052

[B88] Williams PH Thorp R Richardson L Colla S (2014) Bumble Bees of North America: An Identification Guide. Princeton University Press, Princeton, 208 pp.

