# Supplementary material for: An Early Miocene bumble bee from northern Bohemia (Hymenoptera, Apidae)
Source: Zookeys. 2017 Oct 19;(710):43–63. doi: 10.3897/zookeys.710.14714 (PMC5674177; doi:10.3897/zookeys.710.14714)
Supplement: Supplementary material 1 — Table S1 [file zookeys-710-043-s001.docx]

**Table S1.** First dataset for geometric morphometric analyses encompassing 988 specimens from 234 species, 141 genera, 52 tribes, 18 subfamilies, and seven families of Anthophila (Apoidea). All included groups have three submarginal cells. N1= number of species; N2 = number of specimens.

| **Family** | **Subfamily / Clade** | **Tribe** | **N1** | **N2** |
| --- | --- | --- | --- | --- |
| Andrenidae | Andreninae | Andrenini | 4 | 20 |
|  |  | Euherbstiini | 3 | 20 |
|  | Oxaeinae |  | 4 | 20 |
|  | Panurginae | Melitturgini | 4 | 20 |
|  |  | Nolanomelissini | 1 | 10 |
|  |  | Protandrenini | 5 | 20 |
| Apidae | “Non-parasitic Apidae” | Ancylaini | 7 | 20 |
|  |  | Apini | 4 | 20 |
|  |  | Bombini | 4 | 20 |
|  |  | †Electrapini | 5 | 6 |
|  |  | Centridini | 4 | 20 |
|  |  | Emphorini | 4 | 20 |
|  |  | Eucerini | 4 | 20 |
|  |  | Euglossini | 4 | 20 |
|  |  | Exomalopsini | 6 | 20 |
|  |  | †Melikertini | 3 | 3 |
|  |  | Tapinotaspidini | 5 | 20 |
|  |  | Tetrapediini | 5 | 20 |
|  | “Parasitic Apidae” | Anthophorini | 6 | 20 |
|  |  | Brachynomadini | 5 | 20 |
|  |  | Ericrocridini | 4 | 20 |
|  |  | Epeolini | 7 | 20 |
|  |  | Isepeolini | 4 | 20 |
|  |  | Melectini | 6 | 20 |
|  |  | Nomadini | 4 | 20 |
|  |  | Osirini | 5 | 20 |
|  |  | Protepeolini | 4 | 20 |
|  |  | Rhathymini | 3 | 20 |
|  | Xylocopinae | Ceratinini | 4 | 20 |
|  |  | Manuelinii | 3 | 20 |
|  |  | Xylocopini | 4 | 20 |
| Colletidae | Callomelittinae |  | 3 | 20 |
|  | Colletinae | Colletini | 4 | 20 |
|  | Neopasiphaeinae |  | 4 | 20 |
|  | Diphaglossinae | Caupolicanini | 7 | 20 |
|  |  | Diphaglossini | 3 | 20 |
|  |  | Dissoglottini | 3 | 20 |
| Halictidae | Halictinae | Augochlorini | 4 | 20 |
|  |  | Caenohalictini | 5 | 20 |
|  |  | Halictini | 5 | 20 |
|  |  | Sphecodini | 5 | 20 |
|  |  | Thrinchostomatini | 4 | 20 |
|  | Nomiinae |  | 4 | 20 |
|  | Nomioidinae |  | 5 | 20 |
|  | Rophitinae | Conanthalictini | 6 | 20 |
|  |  | Penapini | 4 | 19 |
|  |  | Rophitini | 6 | 20 |
|  |  | Xeralictini | 3 | 20 |
| Megachilidae | Fideliinae | Fideliini | 3 | 20 |
| Melittidae | Meganomiinae |  | 6 | 20 |
|  | Melittinae | Melittini | 6 | 20 |
| Stenotritidae |  |  | 8 | 19 |
